# Multimodal Machine Learning Approach for Diagnosing Atopic Dermatitis

**DOI:** 10.12688/f1000research.169102.2

**Published:** 2026-02-09

**Authors:** Alida Widiawaty, Wresti Indriatmi, Wisnu Jatmiko, Endi Novianto, Aria Kekalih, Hendra Gunawan, Pramudita Satria Palar, Muhammad Febrian Rachmadi, Sherly Dermawan, Tengku Laras Malahayati, Alif Wicaksana Ramadhan

**Affiliations:** 1 Faculty of Medicine, Universitas Riau, Pekanbaru, Riau, Indonesia; 2 Medical Science, Doctoral Study Program, Universitas Indonesia, Jakarta Pusat, Jakarta, Indonesia; 3 Department of Dermatology and Venereology, Universitas Indonesia, Jakarta Pusat, Jakarta, Indonesia; 4 Faculty of Computer Science, Universitas Indonesia, Depok, West Java, Indonesia; 5 Occupational Medicine, Universitas Indonesia, Jakarta Pusat, Jakarta, Indonesia; 6 Department of Dermatology and Venereology, Universitas Padjadjaran, Bandung, West Java, Indonesia; 7 Faculty of Mechanical and Aerospace Engineering, Institut Teknologi Bandung, Bandung, West Java, Indonesia

**Keywords:** Atopic dermatitis, Multimodal Artificial Intelligence (AI), ResNet50, MPNet, Dermatology diagnosis, Clinical decision support, Machine learning, Explainable AI (XAI)

## Abstract

**Background:**

Atopic dermatitis (AD) is a prevalent, chronic inflammatory skin disease with diverse clinical presentations, often overlapping with other dermatoses. Its diagnosis remains largely dependent on clinical expertise, leading to variability and limited diagnostic accuracy, particularly among general practitioners. This study aimed to develop and evaluate a multimodal artificial intelligence (AI) model that integrates lesion image analysis and structured anamnesis to improve AD diagnosis.

**Methods:**

This diagnostic study was conducted in two phases: Phase 1 used retrospective data from 2021–2024, and Phase 2 involved prospective external validation from multiple hospitals in 2025. Patients with AD or related skin conditions were included, with diagnoses based on AAD 2014 criteria. Multimodal fusion combined ResNet50-extracted image features and MPNet-based anamnesis text features using a late fusion model. This approach mimics clinical reasoning by integrating visual and contextual clinical information to classify cases as AD or non-AD.

**Results and Discussion:**

The multimodal AI model integrating ResNet50 (image) and MPNet (anamnesis) achieved 98.28% accuracy in classifying AD vs non-AD, outperforming image-or text-only models. It offers clinical advantages by mimicking physician reasoning, improving diagnostic consistency, reducing subjectivity, and enabling mass triage. However, real-world generalizability remains a challenge due to limited training diversity, potential language constraints (Bahasa Indonesia), and narrow differential diagnoses. External validation and explainable AI (XAI) are critical for broader application. Despite limitations, the model aligns with emerging literature, showing multimodal AI can approach or surpass expert-level performance in dermatological diagnosis when rigorously validated.

**Conclusions:**

The multimodal ResNet50-MPNet model shows near-perfect accuracy in diagnosing AD by mimicking clinician reasoning. It offers consistent, holistic assessment but requires external validation and improved interpretability for clinical adoption. Continued AI-clinician collaboration is vital to translating this promising technology into real-world dermatological care.

## Introduction

Atopic dermatitis (AD) is a chronic and relapsing inflammatory skin disease frequently encountered in both children and adults. Its prevalence is estimated to be 15–20% in children and 10% in adults. ^1– 3^ The onset typically occurs before the age of five, underscoring the importance of timely and accurate diagnosis to prevent complications and improve quality of life. ^4– 6^ Clinically, AD is characterized by severe pruritus and xerosis, and is often associated with allergic comorbidities such as asthma and allergic rhinitis. ^5, 7^ The complexity of its pathogenesis—encompassing genetic, immunologic, and environmental factors—along with diverse clinical presentations can hinder accurate diagnosis. ^8– 10^ Morphologically, AD may mimic other dermatoses (e.g., psoriasis vulgaris, contact dermatitis, nummular dermatitis), leading to potential misdiagnosis if not thoroughly evaluated. ^11– 13^

Another diagnostic challenge is the high inter-clinician variability. Diagnosis of AD currently relies on the clinician’s expertise through careful anamnesis and physical examination. This conventional approach is subjective, resulting in variable diagnostic accuracy, especially among general practitioners. Previous studies report diagnostic accuracy for skin diseases by general practitioners to range from 24% to 70%, markedly lower than that of dermatologists. ^14^ This variation in expertise and experience leads to diagnostic inconsistencies and may result in inappropriate or prolonged treatment. Even standardized severity scoring tools (e.g., SCORAD or EASI) show interobserver disparities due to the subjectivity in assessing certain clinical elements. This condition underscores the need for a more reliable and consistent diagnostic method for AD. ^13, 15^

To address these diagnostic challenges, artificial intelligence (AI) presents a promising solution. Advances in AI, particularly deep learning (DL), offer new opportunities in dermatology to enhance diagnostic accuracy and consistency. Research on AI-based tools for diagnosing inflammatory skin diseases has demonstrated significant potential. ^16– 18^ Various Convolutional Neural Network (CNN)-based algorithms have successfully recognized and classified skin lesions with high accuracy, comparable to that of dermatologists. ^13^

Wu et al. (2020) developed a DL model using EfficientNet-b4 to classify psoriasis and AD from lesion images, achieving accuracy, sensitivity, and specificity rates above 90%. Maron et al. (2019) showed that CNNs systematically outperformed 112 dermatologists in multi-class skin lesion classification, highlighting the potential of DL in dermatological diagnostics. ^19^ Dautovic et al. applied an artificial neural network (ANN) using nine clinical parameters to diagnose AD in both healthy individuals and AD patients. Yang et al. also utilized DL to recognize dermoscopic images of psoriasis and inflammatory diseases such as dermatitis, achieving a sensitivity of 73%. ^20^

Despite these promising results, several limitations persist—particularly the lack of clinical context. Most current models are trained on images alone, neglecting additional clinical information that could enhance diagnostic decision-making. ^21^ Such single-modality approaches risk overlooking important patient context that clinicians routinely consider in clinical practice. In fact, anamnesis and clinical information—such as patient age, chief complaints, history of atopy, lesion distribution, and family medical history—contain valuable insights that can aid in distinguishing AD from its differential diagnoses and provide a more comprehensive assessment of the patient’s condition. Fundamentally, physicians establish a diagnosis based on a combination of history taking and physical examination.

The fact that only a few multimodal AI models are currently available in dermatology—especially for inflammatory skin diseases such as AD—reveals a significant research gap. Integrating clinical data into AI models has the potential to bridge this disparity, in line with the current medical trend of leveraging diverse patient data sources to enhance diagnostic accuracy. Multimodal AI approaches represent a highly promising avenue for achieving more comprehensive and accurate diagnoses of complex conditions such as AD (Yu et al., 2025). ^13, 22, 23^

This study aims to develop and evaluate an AI model with a multimodal approach, combining DL-based clinical image analysis with structured textual anamnesis using a transformer-based language model. Specifically, the architecture employs a 50-layer Residual Network (ResNet50) for image feature extraction and a Masked and Permuted Pre-training for Language Understanding (MPNet) for textual feature extraction. ResNet50 is a widely used CNN model with proven success in both medical and general image classification. ^24^ Its architecture introduces skip connections that help the network learn deep image features without losing critical information. In dermatology, ResNet50 has demonstrated high accuracy—for example, achieving 90% on the ISIC 2018 dataset and 95.8% on the PH2 dataset for skin disease detection. ^25^

MPNet is a transformer-based NLP model designed to produce rich contextual sentence representations. Combining the strengths of BERT and XLNet, MPNet effectively captures complex language patterns and generates high-dimensional text embeddings. In this study, MPNet processes structured anamnesis data (e.g., chief complaints, subjective symptoms, history of allergies, family medical history, and others) into numerical text features. The integration of MPNet ensures that relevant non-visual clinical information is appropriately incorporated into the model.

## Methods

This diagnostic study was conducted in two phases. Phase 1 focused on the development of the AI model. This phase comprised several stages. The first stage involved the collection of medical data through retrospective review of hospital medical records from 2021 to 2024. Data included clinical information and skin lesion images of patients diagnosed with AD, psoriasis vulgaris (PV), chronic lichen simplex (CLS), nummular dermatitis (ND), and contact dermatitis (CD), as diagnosed by board-certified dermatologists. Clinical information incorporated into the text data for non-AD cases comprised sex, age, chief complaint with onset, exposure history to allergens or irritants, source of infection, current triggering factors, disease duration, lesion location, past medical history, and family history. For AD cases, additional inputs included major and minor diagnostic criteria. In the second stage, images were pre-labeled with relevant clinical information. The third stage involved training the machine learning (ML) model to identify discriminative features and characteristics that differentiate AD from non-AD ( Figure 2).

**Figure 1.**
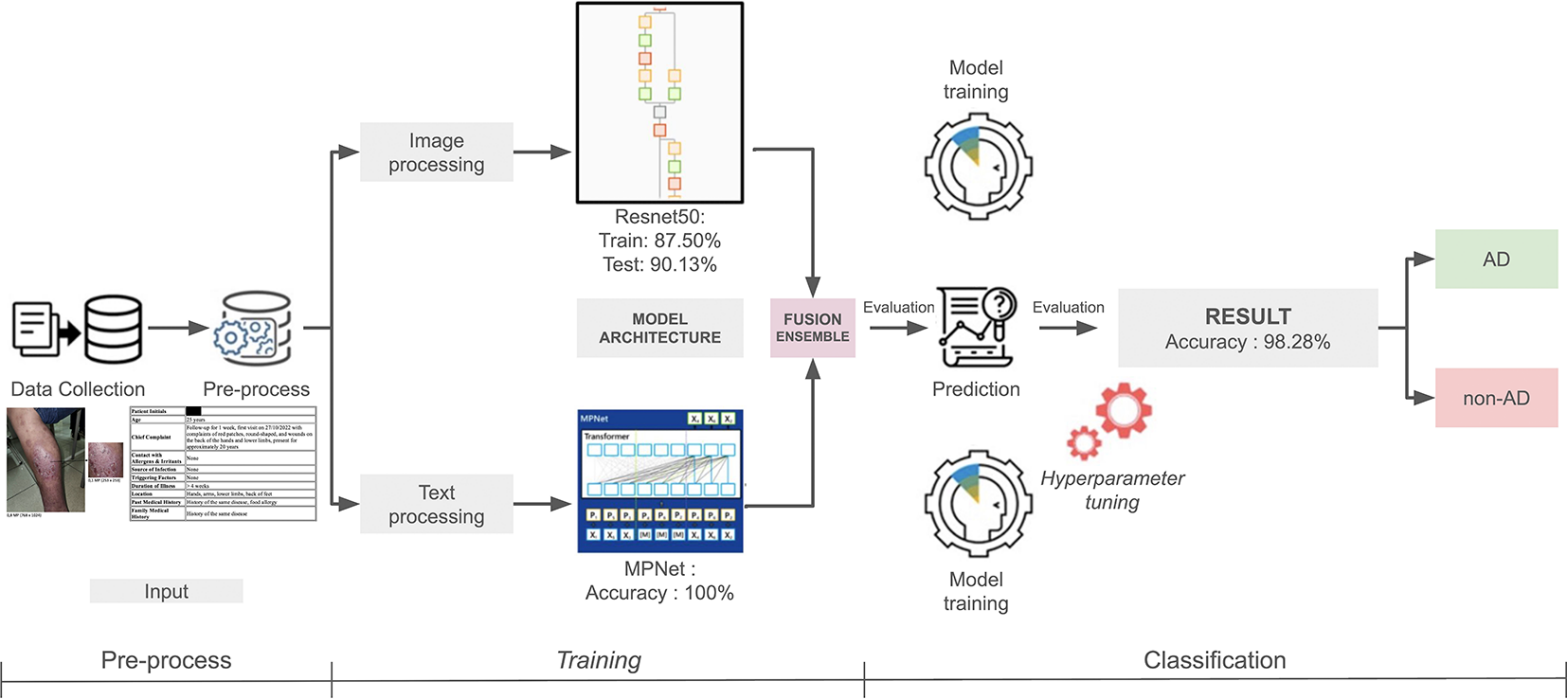
Multimodal model algorithm flowchart.

**Figure 2.**
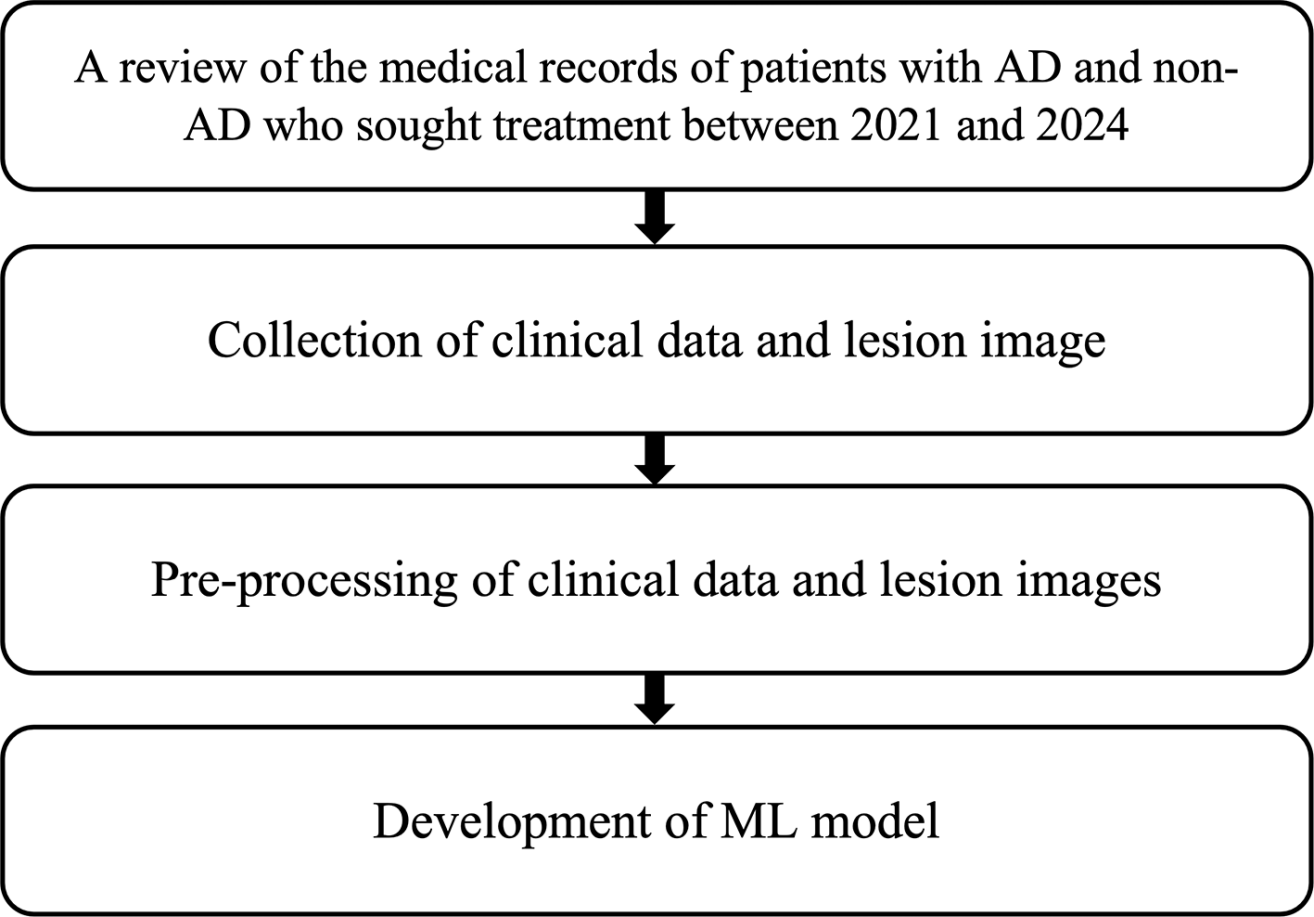
Research flow – Phase 1.

**Figure 3.**
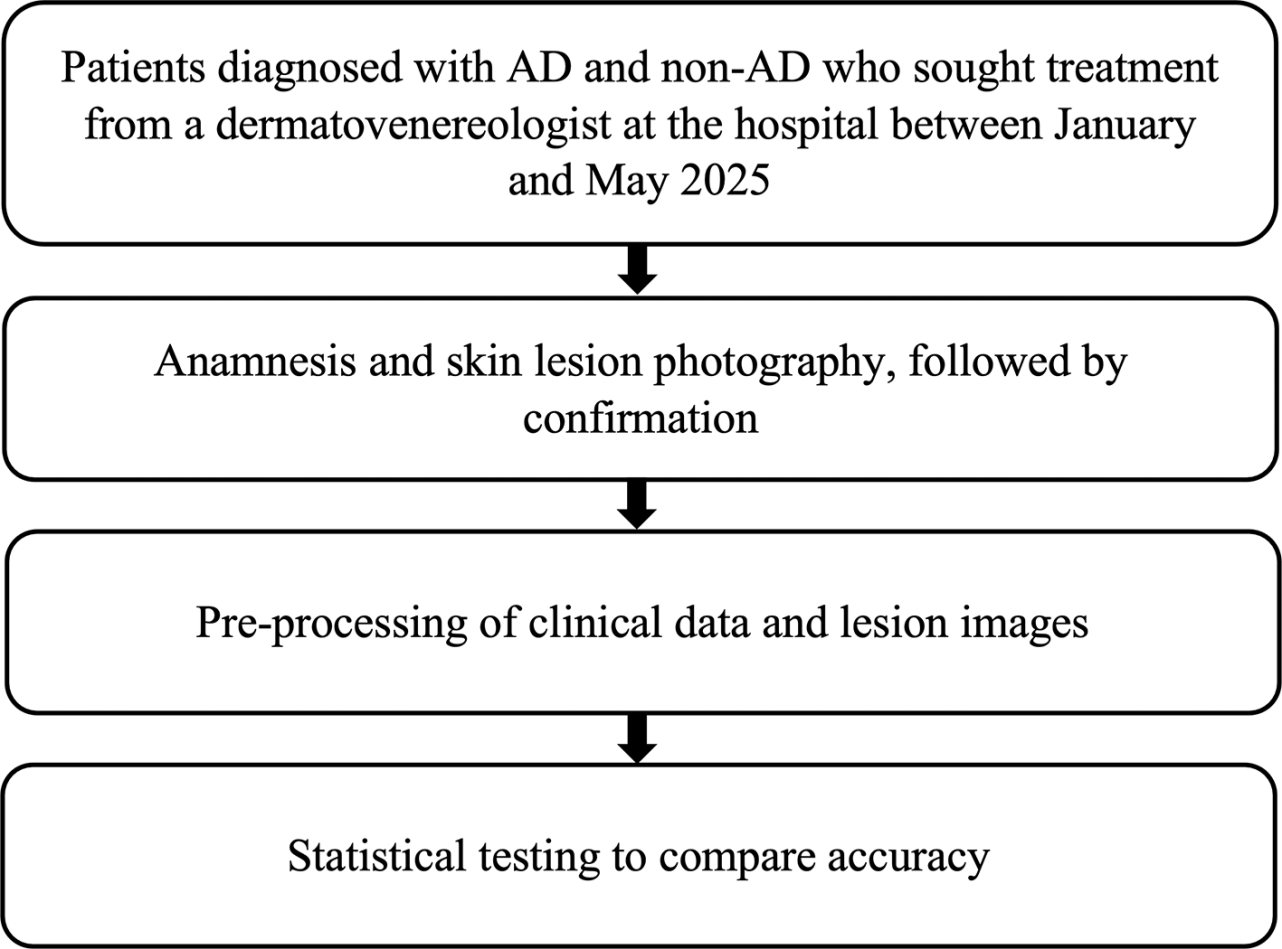
Research flow – Phase 2.

Phase 2 was a multicenter validation study designed to evaluate the generalizability of the ML model trained in Phase 1. Clinical data and lesion images were collected from patients diagnosed with AD or non-AD skin conditions who visited board-certified dermatologists at various hospitals between January and May 2025. In this phase, dermatologists performed complete clinical interviews, and all clinical data were recorded using a structured form ( Figure 3).

In both Phases 1 and 2, skin lesion images were captured using mobile phones owned by the examining physicians. All participants formed a consecutive and provided written informed consent and agreed to the use and publication of their anonymized data, including medical history and lesion images. For minor participants, written consent was obtained from their legal guardians. Ethical approval was obtained from the Ethics Committee of the Faculty of Medicine, Universitas Indonesia–Cipto Mangunkusumo Hospital. The diagnosis of AD was established based on the criteria outlined in the American Academy of Dermatology (AAD) guidelines for the diagnosis and assessment of AD 2014. Two board-certified dermatologists, each with over 20 years of clinical experience in inflammatory dermatoses, independently annotated all images and texts, achieving 100% agreement. As complete agreement was achieved across all images and texts, all annotated photo descriptions were retained for analysis. In cases of discrepant ratings, the corresponding images and associated clinical data would have been excluded from the dataset. The learning outcome was binary: AD or non-AD. All clinical images and patient data were archived systematically. The sample size was calculated using the formula for diagnostic studies, resulting in a minimum requirement of 346 participants for the AD group and 138 for the non-AD group.

Multimodal fusion was implemented by integrating visual features from ResNet50 and textual features from MPNet prior to final prediction. A late fusion approach was used, in which feature vectors from both modalities were concatenated and passed through a classification layer to determine the diagnosis. This method leverages the strengths of both modalities: images provide morphological and distributional characteristics of lesions, while anamnesis text offers clinical context such as chronic pruritus, personal or family history of atopy, and comorbid asthma. The combined approach is expected to reach above 90% accuracy and emulate the way dermatologists diagnose—by integrating visual inspection with the patient’s clinical information.

## Materials

The characteristics of subjects with and without AD are presented in Table 1. In Phase 1, a total of 926 AD samples and 697 non-AD samples were collected, while Phase 2 yielded 525 AD samples and 663 non-AD samples. The dataset was imbalanced with respect to the number of samples per class. To mitigate this, stratified splitting was applied to preserve class proportions across training, validation, and test sets. Model performance was evaluated using weighted precision, recall, and F1-score to account for class imbalance, in addition to overall accuracy.

**Table 1.** Demographic and clinical characteristics of phase 1 and phase 2 study subjects.

Characteristics		Phase 1				Phase 2			
		AD		Non-AD		AD		Non-AD	
		n	%	n	%	n	%	n	%
Age Group	Toddler: 0–5 years	189	20.41	0	0	69	13.14	3	0.45
	Child: 5–11 years	125	13.50	4	0.57	98	18.67	32	4.83
	Early adolescent: 12–16 years	107	11.56	20	2.87	43	8.19	11	1.66
	Late adolescent: 17–25 years	199	21.49	60	8.61	89	16.95	86	12.97
	Young adult: 26–35 years	107	11.56	192	27.54	77	14.67	128	19.31
	Middle-aged adult: 36–45 years	109	11.77	115	16.50	67	12.76	84	12.67
	Early elderly: 46–55 years	73	7.89	232	33.29	41	7.81	142	21.42
	Late elderly: 56–65 years	15	1.61	74	10.62	12	2.29	109	16.44
	Senior: > 65 years	2	0.21	0	0	29	5.52	68	10.26
Sex	Male	460	49.68	256	36.73	242	46.10	313	47.21
	Female	466	50.32	441	63.27	283	53.90	350	52.79
Duration of illness	1 week	31	3.35	16	2.30	18	3.43	58	8.75
	2 weeks	15	1.62	0	0	38	7.24	50	7.54
	3 weeks	0	0	2	0.29	0	0	0	0
	4 weeks	34	3.67	53	7.60	23	4.38	14	2.11
	> 4 weeks	846	91.36	626	89.81	446	84.95	541	81.6

The findings indicate that AD was most frequently observed in infants and children, accounting for 45.45% of cases in Phase 1 and 40% in Phase 2. Its prevalence declined with increasing age, with only 0.21% of cases occurring in individuals over 65 years old. These results are consistent with the known epidemiology of AD, which typically begins in childhood or adolescence. AD most commonly manifests in infancy and early childhood, with approximately 60% of cases developing before the age of one, and nearly all cases occurring before the age of five. ^26^

### Image

The dataset used in this study consists of images of skin lesions from patients diagnosed with AD as well as patients suffering from other skin conditions whose lesions visually resemble those found in AD cases. To perform a supervised classification task, we categorized the images into two distinct classes: AD (representing AD lesions) and Non-AD (representing non-AD lesions). In order to enhance model generalization and robustness, we applied extensive data augmentation prior to training. Each original image was augmented five times using the Albumentations library to produce image sets with various transformations. These included horizontal and vertical flipping, random adjustments to brightness and contrast, rotations of up to 30 degrees, mild Gaussian blurring, and the addition of Gaussian noise. After augmentation, all images were resized to a standardized dimension of 224 × 224 pixels. Furthermore, pixel values were normalized using the ImageNet mean [0.485, 0.456, 0.406] and standard deviation [0.229, 0.224, 0.225] to match the input expectations of the pretrained models. For the classification task, we evaluated two different DL architectures: ResNet50 and Vision Transformer (ViT). Each model’s final classification layer was adapted to produce outputs corresponding to the two target classes, AD and Non-AD. For the record, ViT was included for exploratory comparison only. Given the limited dataset size, the ViT model was not expected to reach optimal performance, as transformer-based architectures typically require substantially larger training datasets. Therefore, ViT results were not used as a basis for direct performance comparison with CNN-based models.

Training was conducted using the PyTorch DL framework, which is widely used in the field of computer vision research due to its flexibility and high performance. Since the dataset used in this study is relatively small in scale, which is a common situation in the medical field where obtaining labeled images can be challenging, it becomes crucial to adopt an evaluation strategy that maximizes the use of available data while still providing a reliable estimate of the model performance. To address this, we employed the use of K-fold Cross Validation.

Rather than splitting the dataset into a single training and testing group, the cross-validation approach divides the dataset into ten equal parts, or “folds.” In each round of training, the model is trained using nine of these folds and evaluated on the remaining one. This process is repeated ten times, with each fold taking a turn as the validation set. At the end of this procedure, the results from all ten rounds are averaged to provide a comprehensive assessment of the model’s performance. This technique reduces the risk of the evaluation being biased by the specific choice of training or testing data and is particularly important when dealing with medical datasets, where the number of samples can be limited. It offers a favorable balance between model accuracy and computational efficiency.

During training, we also implemented an optimization algorithm called Adam, which automatically adjusts the learning rate during training to improve the model’s ability to learn from data. To further refine the training process, we applied a strategy called learning rate scheduling, where the learning rate was reduced gradually over time to allow the model to converge more smoothly. In addition, we implemented early stopping, a technique where the training process is halted if the model’s performance on validation data does not improve after a few consecutive training rounds. This prevents the model from overfitting, or memorizing the training data too much, and helps ensure that it learns patterns that are generalizable to unseen data.

Throughout the training and evaluation process, we monitored several key metrics to assess model performance. These included accuracy, which measures how often the model’s predictions matched the correct labels; precision, which indicates how many of the predicted DA cases were actually correct; recall, which measures how many actual DA cases the model successfully identified; and F1-score, which balances precision and recall into a single number to give an overall sense of the model’s effectiveness. By considering these multiple aspects, we aimed to evaluate not just whether the model made correct predictions overall, but also whether it was good at correctly identifying cases of AD without missing too many or falsely predicting AD where there was none.

After completing the full training and evaluation pipeline, ResNet50 demonstrated superior performance, achieving an accuracy of 0.8750, compared to 0.6013 for the ViT. ResNet50 consistently demonstrated superior performance across all key metrics, including higher accuracy, precision, recall, and F1-score ( Table 2). In particular, ResNet50 exhibited strong stability during training and converged more rapidly. These results suggest that convolutional architectures with residual connections, such as ResNet, are particularly well-suited for medical image classification tasks such as skin lesion analysis, where fine-grained texture analysis is the highlight.

**Table 2.** Key performance metrics of Resnet50.

Model	Accuracy	Precision	Recall	F1-Score
Resnet50	0.8750	0.8809	0.8750	0.8728

### Text

The dataset used in this study consists of anamnesis text of patients whose diagnosed with AD as well as patients suffering from other skin conditions whose lesions visually resemble those found in AD cases. Clinical text inputs were derived from dermatologist notes within medical records during Phase 1, and from patient anamnesis recorded in structured forms during Phase 2. The text in the dataset are written in Bahasa Indonesia. To perform a supervised classification task, we categorized the anamnesis into two classes, the AD and Non-AD. The anamnesis contains several parameters such as patient age, gender, symptoms, contact history, etc. Those parameters were then configured to follows a template as follows:

Pasien dengan jenis kelamin: <gender>
usia: <age>
klasifikasi usia: <age_classification>
Keluhan utama dan onset: <main_onset_symptoms>
Riwayat kontak dengan bahan alergen atau iritan: <irritant_allergent_contact_hisotry>
Sumber infeksi: <infection_source>
Faktor pencetus penyakit saat ini: <current_disease_cause>
Lama sakit: <disease_duration>
Lokasi lesi: <lesion_location>
Kriteria mayor: <major_criterion>
Kriteria minor: <minor_criterion>
Riwayat penyakit dahulu: <old_historical_disease>
Riwayat penyakit keluarga: <familiy_historical_disease>



By generating text following this template, we obtained a string as the model input to be classified by the proposed method. After we obtain the full text as model input, we need to do pre-processing to the text before feeding it to the model. In this text processing task, the preprocessing part was done by using a tokenizer. A tokenizer is a tool or process used in natural language processing (NLP) to break down a text into smaller units, called tokens. These tokens can be words, subwords, or characters, depending on the tokenization method used. The purpose of tokenization is to transform raw text into a more manageable and structured form that can be analyzed or fed into ML models.

In this study we used a pre-trained model, MPNet which is a state-of-the-art transformer model that was introduced as a variant of BERT (Bidirectional Encoder Representations from Transformers) and other transformer-based models like RoBERTa and ALBERT. MPNet improves upon BERT’s pretraining strategy by using a more sophisticated approach for handling masked tokens and learning better dependencies between tokens in a sequence. The tokenizer used in MPNet is based on the WordPiece tokenization method, which is also used in BERT and similar transformer models.

The output of this MPNet pre-trained model is 768 sized vectors. To do classification task, we added some dense networks downstream the MPNet architecture and formed a single output classification. The model was created using PyTorch DL framework, which is widely used in the field of computer vision research due to its flexibility and high performance. For the training process, we splitted the dataset into training and validation parts with a ratio of 8:2. After the training process, the fine-tuned MPNet model obtained an accuracy of 100% in both validation and training data.

### Multimodal

The multimodal approach in this study integrates two powerful models: ResNet50 for visual data and MPNet for textual data, to enhance classification performance by leveraging both image and text information. ResNet50, a deep CNN, is employed to process the images of skin lesions. These images are first preprocessed through augmentation, resizing, and normalization, ensuring compatibility with the input requirements of ResNet50. The model then generates a feature vector from the image, capturing the essential visual characteristics of the skin lesion, which represents the image data for subsequent processing.

In parallel, MPNet, a transformer-based model, is applied to the textual data consisting of patients’ anamnesis information. MPNet tokenizes the text and generates a fixed-size vector (768-dimensional), which encodes the relationships and context between the words. This representation is essential for understanding the nuanced medical history of the patients, such as symptoms, age, and other relevant factors. MPNet’s ability to model contextual dependencies and generate more accurate token representations provides an edge over traditional models like BERT, especially when dealing with complex and diverse medical texts.

Once ResNet50 and MPNet processed their respective modalities and generated modality-specific feature embeddings, the resulting vectors were concatenated to form a unified multimodal representation. This fusion strategy enables the integration of fine-grained visual information from images with rich contextual data derived from clinical text. The image and text encoders were trained independently, and their parameters were fixed during multimodal training. The concatenated feature vector was subsequently passed through several fully connected layers, which were trained as a separate classifier to learn the most discriminative multimodal patterns and to distinguish between AD and non-AD cases. No end-to-end joint optimization across modalities was performed.

## Results

### Clinical implications, strengths, and limitations of the multimodal AI model

The image-only model achieved an accuracy of 87.50% on the training set and 90.13% on the test set. Key performance metrics, including accuracy, precision, recall, and F1 score are presented in Table 2. The text-only model achieved 100% accuracy on both the training and validation sets. Following the fusion of both modalities, the overall accuracy increased significantly to 98.28% ( Figure 1).

The multimodal AI approach employed in this study offers several advantages. First, the AI model can consistently analyze hundreds of microscopic visual features and textual patterns without fatigue, enabling the potential for high accuracy and sensitivity across diverse cases. In fact, numerous studies have demonstrated that AI performance in dermatological diagnosis can match or even surpass that of dermatology specialists for certain specific tasks. ^27^ For instance, a recent meta-analysis by Salinas et al. (2024) on skin cancer diagnosis reported AI algorithms achieving a sensitivity of 87% and specificity of 77%, comparable to expert dermatologists (sensitivity 84%, specificity 74%). ^28^ In the context of AD, the ResNet50 model, when specifically trained, has outperformed conventional models and approached expert-level performance in assessing AD severity. ^29^ By integrating anamnesis data, AI can also account for factors typically gathered through patient interviews, thereby allowing for more clinically informed decision-making.

AI systems can also process multiple cases simultaneously, supporting mass screening or triage workflows, thereby allowing physicians to prioritize complex cases. Moreover, multimodal AI reduces subjectivity: decisions are derived from patterns learned across hundreds of examples, rather than individual intuition, which can vary among physicians. With a reported accuracy of 98.28%, this system has strong potential as a reliable diagnostic aid, offering second opinions to clinicians and enhancing general practitioners’ confidence in correctly identifying AD. ^30^

Nevertheless, several important considerations must be addressed. First, the model’s outstanding performance in a controlled setting may not necessarily reflect its effectiveness in real-world clinical practice. Generalizability remains a common challenge in AI models, as they may learn patterns specific to a particular hospital’s dataset, potentially resulting in reduced accuracy when applied to different patient populations or images captured with other camera devices. While 10-fold cross-validation provides a comprehensive internal evaluation, external validation using data from independent clinics is essential to ensure the model is not overfitting. The high accuracy of 98.28% raises a concern that the model may have been overtrained on a limited dataset. In this study, external validation was conducted using datasets from multiple hospitals to ensure that the model maintains reliable and consistent performance. In future work, explainable AI (XAI) techniques could be employed to enhance interpretability, allowing the model’s decision-making process to be visually explained and verified against clinical reasoning. ^31, 32^

Another key issue is interpretability. Although AI can achieve high levels of accuracy, it remains limited in its ability to explain the rationale behind a given prediction. Physicians, by contrast, can justify a diagnosis based on observable clinical features (e.g., “there is excoriation due to intense itching, a typical distribution pattern on the infant’s cheeks, and a positive family history of atopy, consistent with AD”). AI models require XAI techniques to offer similar justifications. Without such interpretability, trust in AI-generated recommendations—both among physicians and patients—may be diminished. Moreover, broader clinical context is often necessary for final decision-making. Physicians may rely on direct physical examination (e.g., palpating the skin texture), additional diagnostic tests, or further clinical history. AI models, on the other hand, can only process the limited input they are provided (i.e., images and structured textual data), making their reasoning less flexible than that of physicians. Clinicians can pose follow-up questions, revise diagnostic hypotheses in real time, and manage atypical or complex cases not represented in the training data—for example, a patient presenting with two concurrent skin conditions.

Finally, physicians possess critical strengths in empathy and clinical ethics—for example, delivering a diagnosis and treatment plan in a psychologically appropriate and compassionate manner. This human element lies beyond the scope of AI models. Therefore, AI should be viewed as a complementary tool that supports physicians, rather than as a full replacement. AI is intended to serve as a decision support system, not a substitute for clinical judgment. Collaborative use of AI and physicians has been shown to improve diagnostic accuracy compared to either working alone. ^33^ In practice, AI can provide a rapid second opinion, while the final decision and any necessary contextual adjustments remain the responsibility of the physician.

## Discussion

### Accuracy of 98.28% and its relevance in current literature

The final accuracy of 98.28% (95% confidence interval = 96.93 - 99.65), sensitivity of 99.71% (95% confidence interval = 99.14 - 100), specificity of 97.21% (95% confidence interval = 95.01 - 99.41), Negative Predictive Value (NPV) 99.5% and Positive Predictive Value (PPV) 98.3% achieved by the multimodal ResNet50 and MPNet model in distinguishing between AD and non-AD cases represents an exceptionally high performance ( Table 3). To assess its relevance, this result must be compared with recent studies in the field of computer-aided dermatological diagnosis. In general, AI models for skin lesion classification typically achieve accuracies above 80–90%. For example, Wu et al., (2020) developed and validated an image-based DL model, AIDDA, using the EfficientNet-b4 convolutional neural network architecture to automate the diagnosis of common inflammatory skin diseases, including psoriasis, eczema, and AD. Trained on a dataset of 4,740 expert-labeled clinical images, the model achieved high diagnostic performance, with an overall accuracy of 95.8%, sensitivity of 94.4%, and specificity of 97.2%.

**Table 3.** Diagnostic performance of the multimodal ResNet50–MPNet model.

Metric	Value (%)	95% CI
Accuracy	98.28	96.93-99.65
Sensitivity	99.71	99.14-100
Specificity	97.21	95.01-99.41

Therefore, the 98.28% accuracy achieved in this study warrants attention as a highly promising result. It highlights the potential advantage of a multimodal approach. By incorporating additional clinical data, the model may resolve ambiguities that remain when relying on image analysis alone, allowing for near-perfect classification across samples. Other multimodal studies have also reported strong performance. Adebiyi et al. (2024), for instance, integrated lesion images with patient metadata and achieved an area under the curve (AUC) of 0.94 (94%) on the HAM10000 dataset—an improvement over image-only models. ^34^ In addition, recent developments in transformer-based fusion approaches, such as TFormer, have been specifically designed to deeply integrate multimodal information and have demonstrated enhanced diagnostic accuracy across various types of skin lesions. ^17^

Nevertheless, the scientific community emphasizes the necessity of validating AI models in real-world clinical scenarios. Burlando et al. (2024), in a recent meta-analysis, stressed the importance of external validation across diverse populations and collaborative decision-making between AI and clinicians. ^28^ Near-perfect accuracy may indicate model excellence, but also raises the concern of potential data leakage or bias. For instance, if structured anamnesis texts contain inadvertently predictive features (e.g., specific keywords unique to AD cases), the model might overfit to non-clinical artifacts. Thus, transparent methodological reporting—such as details on 10-fold cross-validation and feature importance analyses—is essential for interpreting these results responsibly.

The 98.28% accuracy is consistent with previous studies reporting that advanced DL models, particularly those utilizing multimodal inputs, can achieve diagnostic performance comparable to or exceeding that of experienced clinicians in specific tasks. ^19^ This parallels recent innovations in digital dermatology, such as the PanDerm foundation model, trained on millions of multimodal images, which outperformed dermatologists in early melanoma detection. ^35^ Similarly, generative models like SkinGPT-4 (2024) now integrate ViT with large language models to generate diagnostic reports that approximate physician reasoning. ^36^ In this context, our findings contribute compelling evidence that multimodal AI has the potential to reshape dermatological diagnostics, provided that it is demonstrated to be consistent in larger-scale studies.

Several limitations warrant consideration. First, the model was trained on anamnesis written in Bahasa Indonesia using a language-specific version of MPNet. Although MPNet has multilingual variants, the one applied here may not generalize effectively to English or other languages. Moreover, differences in documentation style (e.g., brief vs. narrative notes) could also impact model performance. Future implementations should consider language adaptation or employ inherently multilingual models for broader applicability. ^37^

Second, the differential diagnosis in the present study was restricted to four conditions: lichen simplex chronicus, psoriasis vulgaris, nummular dermatitis, and contact dermatitis. In clinical reality, AD mimickers may also include conditions such as scabies, fungal infections, or seborrheic dermatitis, which were not represented in the training data. This raises concerns about generalizability when the model is applied to unfamiliar cases. Expanding the dataset to encompass a broader spectrum of dermatoses, or transitioning to a multiclass classification model, could improve both utility and clinical relevance.

Third, real-world deployment of this system would require regulatory approval, robust documentation of safety and reliability, and integration with electronic health records to ensure seamless clinical workflow. This would necessitate interdisciplinary collaboration among clinicians, engineers, and policy makers. However, to successfully implement AI, key barriers need to be addressed. Efforts should focus on ensuring algorithm transparency and adequate regulation of algorithms. Simultaneously, improving knowledge about AI could reduce the fear of replacement. Fourth, the comparison between CNN-based models and ViT should be interpreted with caution. Transformer-based architectures generally require large-scale datasets to perform optimally, and the relatively small dataset in this study likely constrained ViT performance.

## Conclusion

The multimodal approach combining ResNet50 and MPNet represents a breakthrough in the automated diagnosis of AD. By integrating image and textual data, the model is able to emulate a holistic diagnostic approach similar to that of clinicians, as demonstrated by its near-perfect internal accuracy. Compared to baseline physician assessments, this model offers the potential for greater consistency in diagnosis. However, its adoption in clinical practice must be supported by robust external validation and improved interpretability to gain trust within the medical community. The reported 98.28% accuracy reflects the promise of multimodal AI in dermatology, echoing broader trends in the field toward increasingly sophisticated diagnostic systems. Nonetheless, it is essential to remain critical of the limitations and challenges involved in real-world implementation. With continued advancement in AI research, collaboration between AI and clinicians holds great promise for improving diagnostic accuracy and the overall quality of care, particularly for patients with AD and other dermatologic conditions. ^38, 39^

### Ethical considerations

Ethical approval for the study was obtained from the Health Research Ethics Committee, Faculty of Medicine, Universitas Indonesia–Cipto Mangunkusumo Hospital (certificate number KET-1477/UN2.F1/ETIK/PPM.00.02/2024). All participants provided written informed consent prior to data collection. For minor participants, written consent was obtained from their legal guardians. Ethical approval was obtained from the Ethics Committee of the Faculty of Medicine, Universitas Indonesia–Cipto Mangunkusumo Hospital. Patient data and images were anonymized before use, and no identifiable information was included in the analysis. This diagnostic study did not involve any interventional procedures.

## Data Availability

Figshare: **Multimodal Machine Learning Approach for Diagnosing Atopic Dermatitis**. ^40^ https://doi.org/10.6084/m9.figshare.29925533 The project contains the following underlying data:
• Clinical data of AD and non-AD research samples from phases 1 and 2.xlsx. Figshare: **Multimodal Machine Learning Approach for Diagnosing Atopic Dermatitis**. ^40^ https://doi.org/10.6084/m9.figshare.29925533 The project contains the following underlying data:
• Questionnaires in the form of a Google Form containing clinical data of AD and non-AD patients • Translated and signed informed consent forms obtained from participants prior to lesion photography •
Tables and Figures Figshare: *STARD checklist* for *“* **Multimodal Machine Learning Approach for Diagnosing Atopic Dermatitis** *”* https://doi.org/10.6084/m9.figshare.29925533 Data are available under the terms of the Creative Commons Attribution 4.0 International license (CC-BY 4.0).

## Software availability

**Source code available from:**

• https://github.com/AlidaWidiawaty/multimodal-skin-lesion-classification ^41^
• https://github.com/AlidaWidiawaty/multimodal-dermatitis-classification-anamnesys ^42^

**Archived software availabile from:**

• https://doi.org/10.5281/zenodo.16983448 ^43^
• https://doi.org/10.5281/zenodo.16983460 ^44^

**License:** MIT License

## References

[1] Langan SM Mulick AR Rutter CE : Trends in eczema prevalence in children and adolescents: A Global Asthma Network Phase I Study. *Clin Exp Allergy.* 2023 Mar;53(3):337–352. 10.1111/cea.14276

[2] Hadi HA Tarmizi AI Khalid KA : The Epidemiology and Global Burden of Atopic Dermatitis: A Narrative Review. *Life (Basel).* 2021 Sept 9;11(9):936. 10.3390/life11090936 34575085 PMC8470589

[3] Urban K Mehrmal S Uppal P : The global burden of skin cancer: A longitudinal analysis from the Global Burden of Disease Study, 1990-2017. *JAAD Int.* 2021 Mar;2:98–108. 10.1016/j.jdin.2020.10.013 34409358 PMC8362234

[4] Kelly KA Balogh EA Kaplan SG : Skin Disease in Children: Effects on Quality of Life, Stigmatization, Bullying, and Suicide Risk in Pediatric Acne, Atopic Dermatitis, and Psoriasis Patients. *Children (Basel).* 2021 Nov 16;8(11):1057. 10.3390/children8111057 34828770 PMC8619705

[5] Abdel-Mageed HM : Atopic dermatitis: a comprehensive updated review of this intriguing disease with futuristic insights. *Inflammopharmacol.* 2025 Mar 1;33(3):1161–1187. 10.1007/s10787-025-01642-z 39918744 PMC11914373

[6] Hon KL Chu S Leung AKC : Quality of Life for Children with Allergic Skin Diseases. *Curr. Pediatr. Rev.* 2022;18(3):191–196. 10.2174/1573396317666210901124211 34488587

[7] Jeskey J Kurien C Blunk H : Atopic Dermatitis: A Review of Diagnosis and Treatment. *J. Pediatr. Pharmacol. Ther.* 2024 Dec;29(6):587–603. 10.5863/1551-6776-29.6.587 39659858 PMC11627575

[8] Fyhrquist N Yang Y Karisola P : Endotypes of atopic dermatitis. *J. Allergy Clin. Immunol.* 2025 July;156(1):24–40.e4. 10.1016/j.jaci.2025.02.029 40054744

[9] Criado PR Miot HA Bueno-Filho R : Update on the pathogenesis of atopic dermatitis. *An. Bras. Dermatol.* 2024;99(6):895–915. 10.1016/j.abd.2024.06.001 39138034 PMC11551276

[10] Eichenfield LF Tom WL Berger TG : Guidelines of care for the management of atopic dermatitis: section 2. Management and treatment of atopic dermatitis with topical therapies. *J. Am. Acad. Dermatol.* 2014 July;71(1):116–132. 10.1016/j.jaad.2014.03.023 24813302 PMC4326095

[11] Johansson EK Bergström A Kull I : Prevalence and characteristics of atopic dermatitis among young adult females and males-report from the Swedish population-based study BAMSE. *J. Eur. Acad. Dermatol. Venereol.* 2022 May;36(5):698–704. 10.1111/jdv.17929 35032357 PMC9303811

[12] Napolitano M Fabbrocini G Martora F : Children atopic dermatitis: Diagnosis, mimics, overlaps, and therapeutic implication. *Dermatol. Ther.* 2022 Dec;35(12):e15901. 10.1111/dth.15901 36200594 PMC10078507

[13] Cao F Yang Y Guo C : Advancements in artificial intelligence for atopic dermatitis: diagnosis, treatment, and patient management. *Ann. Med.* 2025 Dec;57(1):2484665. 10.1080/07853890.2025.2484665 40200717 PMC11983576

[14] Tran H Chen K Lim AC : Assessing diagnostic skill in dermatology: a comparison between general practitioners and dermatologists. *Australas. J. Dermatol.* 2005 Nov;46(4):230–234. 10.1111/j.1440-0960.2005.00189.x 16197420

[15] Chopra R Vakharia PP Sacotte R : Relationship between EASI and SCORAD severity assessments for atopic dermatitis. *J. Allergy Clin. Immunol.* 2017 Dec;140(6):1708–1710.e1. 10.1016/j.jaci.2017.04.052 28625808 PMC5723207

[16] Chan S Reddy V Myers B : Machine Learning in Dermatology: Current Applications, Opportunities, and Limitations. *Dermatol Ther (Heidelb).* 2020 June;10(3):365–386. 10.1007/s13555-020-00372-0 32253623 PMC7211783

[17] Zhang Y Xie F Chen J : TFormer: A throughout fusion transformer for multi-modal skin lesion diagnosis. *Comput. Biol. Med.* 2023 May;157:106712. 10.1016/j.compbiomed.2023.106712 36907033

[18] Escalé-Besa A Yélamos O Vidal-Alaball J : Exploring the potential of artificial intelligence in improving skin lesion diagnosis in primary care. *Sci. Rep.* 2023 Mar 15;13(1):4293. 10.1038/s41598-023-31340-1 36922556 PMC10015524

[19] Wu H Yin H Chen H : A deep learning, image based approach for automated diagnosis for inflammatory skin diseases. *Ann Transl Med.* 2020 May;8(9):581. 10.21037/atm.2020.04.39 32566608 PMC7290553

[20] Dautovic A Dondras B Dervisbegovic F : Diagnosis of Atopic dermatitis Using Artificial Neural Network. *IFAC PapersOnLine.* 2022;55(4):51–55. 10.1016/j.ifacol.2022.06.008

[21] Luo N Zhong X Su L : Artificial intelligence-assisted dermatology diagnosis: From unimodal to multimodal. *Comput. Biol. Med.* 2023 Oct;165:107413. 10.1016/j.compbiomed.2023.107413 37703714

[22] Yu Y Jia H Zhang L : Deep multi-modal skin-imaging based information-switching network for skinlesion recognition. *Bioengineering.* 2025;12:282. 10.3390/bioengineering12030282 40150746 PMC11939189

[23] Mohamed EH Abdu N Khalil M : MiSC: A hybrid multi-modal deep learning approach for accurate skin cancer detection. *Multimed. Tools Appl.* 2025 June 4 [cited 2025 Aug 15]. 10.1007/s11042-025-20951-7

[24] Abid MH Ashraf R Mahmood T : Multi-modal medical image classification using deep residual network and genetic algorithm. *PLoS One.* 2023;18(6):e0287786. 10.1371/journal.pone.0287786 37384779 PMC10309999

[25] Musthafa MM Mahesh TR Vinoth Kumar V : Enhanced skin cancer diagnosis using optimized CNN architecture and checkpoints for automated dermatological lesion classification. *BMC Med. Imaging.* 2024 Aug 2;24(1):201. 10.1186/s12880-024-01356-8 39095688 PMC11295341

[26] Kolb L Ferrer-Bruker SJ : Atopic Dermatitis. *StatPearls.* Treasure Island (FL): StatPearls Publishing;2025 [cited 2025 Aug 15]. Reference Source 28846349

[27] Mevorach L Farcomeni A Pellacani G : A Comparison of Skin Lesions’ Diagnoses Between AI-Based Image Classification, an Expert Dermatologist, and a Non-Expert. *Diagnostics (Basel).* 2025 Apr 28;15(9):1115. 10.3390/diagnostics15091115 40361933 PMC12071753

[28] Salinas MP Sepúlveda J Hidalgo L : A systematic review and meta-analysis of artificial intelligence versus clinicians for skin cancer diagnosis. *npj Digit Med.* 2024 May 14;7(1):125. 10.1038/s41746-024-01103-x 38744955 PMC11094047

[29] Maulana A Noviandy TR Suhendra R : Evaluation of atopic dermatitis severity using artificial intelligence. *Narra J.* 2023 Dec;3(3):e511. 10.52225/narra.v3i3.511 38450339 PMC10914065

[30] Park H Woo CY Lim S : Comparisons between a Large Language Model-based Real-Time Compound Diagnostic Medical AI Interface and Physicians for Common Internal Medicine Cases using Simulated Patients. *arXiv.* 2025 [cited 2025 June 19]. Reference Source

[31] Hond AAH de Leeuwenberg AM Hooft L : Guidelines and quality criteria for artificial intelligence-based prediction models in healthcare: a scoping review. *npj Digit Med.* 2022 Jan 10;5(1):2. 10.1038/s41746-021-00549-7 35013569 PMC8748878

[32] Liopyris K Gregoriou S Dias J : Artificial Intelligence in Dermatology: Challenges and Perspectives. *Dermatol Ther (Heidelb).* 2022 Oct 28;12(12):2637–2651. 10.1007/s13555-022-00833-8 36306100 PMC9674813

[33] Verghese A Shah NH Harrington RA : What This Computer Needs Is a Physician: Humanism and Artificial Intelligence. *JAMA.* 2018 Jan 2;319(1):19–20. 10.1001/jama.2017.19198 29261830

[34] Raza A Penuel WR Sumner T : Designing Visual Learning Analytics for Supporting Equity in STEM Classrooms. *arXiv.org.* 2024 [cited 2025 June 19]:1–14. 10.18608/jla.2023.8199 Reference Source

[35] Yan S Yu Z Primiero C : A multimodal vision foundation model for clinical dermatology. *Nat. Med.* 2025 June 6;1–12. 40481209 10.1038/s41591-025-03747-y PMC12353815

[36] Zhou J He X Sun L : Pre-trained multimodal large language model enhances dermatological diagnosis using SkinGPT-4. *Nat. Commun.* 2024 July 5;15(1):5649. 10.1038/s41467-024-50043-3 38969632 PMC11226626

[37] Muennighoff N Tazi N Magne L : MTEB: Massive Text Embedding Benchmark. *arXiv.* 2023 [cited 2025 June 19]. Reference Source

[38] Daneshjou R Kovarik C Ko JM : Towards Realization of Augmented Intelligence in Dermatology: Advances and Future Directions. *arXiv.org.* 2021 [cited 2025 June 19]. Reference Source

[39] Kaczmarczyk R Wilhelm TI Martin R : Evaluating multimodal AI in medical diagnostics. *npj Digit Med.* 2024 Aug 7;7(1):205. 10.1038/s41746-024-01208-3 39112822 PMC11306783

[40] Multimodal Machine Learning Approach for Diagnosing Atopic Dermatitis. *figshare* 2025 [cited 2025 Aug 17]. Reference Source 10.12688/f1000research.169102.2 PMC12914175 41716356

[41] AlidaWidiawaty: AlidaWidiawaty/multimodal-skin-lesion-classification. 2025 [cited 2025 Aug 29]. Reference Source

[42] AlidaWidiawaty: AlidaWidiawaty/multimodal-dermatitis-classification-anamnesys. 2025 [cited 2025 Aug 29]. Reference Source

[43] AlidaWidiawaty: AlidaWidiawaty/multimodal-skin-lesion-classification: v1.0.0. *Zenodo.* 2025 [cited 2025 Aug 29]. Reference Source

[44] AlidaWidiawaty: AlidaWidiawaty/multimodal-dermatitis-classification-anamnesys: v1.0.0. *Zenodo.* 2025 [cited 2025 Aug 29]. Reference Source

