# Supplementary material for: Multimodal Machine Learning Approach for Diagnosing Atopic Dermatitis
Source: F1000Res. 2026 Feb 9;14:952. Originally published 2025 Sep 19. [Version 2] doi: 10.12688/f1000research.169102.2 (PMC12914175; doi:10.12688/f1000research.169102.2)
Supplement: Peer review history

# Reviewer report: Reviewer response for version 2

**DOI:** 10.5256/f1000research.195755.r456887

**Published:** 2026-02-17

**Authors:** Tomasz Krzywicki

**Affiliations:** 1 University of Warmia and Mazury in Olsztyn, Olsztyn, Warmian-Masurian Voivodeship, Poland

I thank the authors for addressing my comments. The article now looks much better. I have no further comments.

Is the work clearly and accurately presented and does it cite the current literature?

Yes

If applicable, is the statistical analysis and its interpretation appropriate?

Partly

Are all the source data underlying the results available to ensure full reproducibility?

No

Is the study design appropriate and is the work technically sound?

No

Are the conclusions drawn adequately supported by the results?

Partly

Are sufficient details of methods and analysis provided to allow replication by others?

No

Reviewer Expertise:

computer vision, deep learning, reinforcement learning, biomedical engineering, parallel processing

I confirm that I have read this submission and believe that I have an appropriate level of expertise to confirm that it is of an acceptable scientific standard.

# Reviewer report: Reviewer response for version 1

**DOI:** 10.5256/f1000research.186388.r446710

**Published:** 2026-01-16

**Authors:** Tomasz Krzywicki

**Affiliations:** 1 University of Warmia and Mazury in Olsztyn, Olsztyn, Warmian-Masurian Voivodeship, Poland

The article concerns the proposal of a solution in the form of a multimodal neural network model supporting skin diagnostics by aggregating clinical information from photos and texts. The idea of aggregation of many modalities in modelling a clinical decision is already well known in the scientific literature, and the authors used in this case the common generic families of neural network architectures: ResNet and BERT.

 While the article itself looks interesting, I have noticed a few flaws that the authors should refer to.

 It is worth specifying at the beginning what kind of clinical information was included in the text data. Was there information that was unobservable from the point of view of the visual skin analysis, which meant that it was not correlated with the information contained in the photos?

 It is worth clarifying what experience the specialists have had in describing the data.

 Is it worth clarifying how the ground truth of the photo descriptions was selected? What would be the procedure if the specialists (with a completely independent evaluation) assigned different ratings to the photos?

 Sections regarding descriptions of dataset characteristics, details of training models and initial data preparation should be moved from "Results" to "Methods" or create a separate chapter, e.g. "Materials". The details of the training do not apply to the results. This chapter should contain the final results.

 Table 2 should be included in the paragraph with the first reference to. In this table, the authors present the result of the comparison of the accuracy of the classification obtained on the ViT and ResNet architectures. It is worth mentioning that these results, with the current data set, should not be summarised with each other. One of the important requirements of the Transformer architecture is a sufficiently large set of training data, where most often the minimum requirements apply to several hundred thousand images, and reasonably, at least a million. With such a small dataset, the ViT architecture is doomed to failure. With such a small dataset, other CNN-based architectures should be compared, e.g. ResNet, Inception and Xception.

 The article lacks a coherent scheme presenting the final multimodal architecture and information about the training. Yes, Figure 3 presents a complete flow from data collection stage to inference. However, there is no information about the final model itself. Were the models that process the image and text learned together with the final classifier, jointly correcting errors? Or is the final classifier a separate model that classifies independent image and text inputs?

 Figures 1 and 2 only provide information about how the quality of the classification indicators changed during the training iterations. In my opinion, they do not contribute anything significant to the results. In addition, Figure 1 shows the features of overfitting model based on the ResNet architecture, which raises serious doubts about the reliability of the results.

 The dataset used in the experiment was unbalanced in terms of the number of photos in decision classes. The authors do not specify how they solved this problem in the context of training and evaluating model results. According to the names of the quality measures, the authors refer to their global versions intended for balanced datasets.

 Figures 4 and 5 are trivial and contribute nothing but to the repetition of the content of the previous paragraphs.

 The final results of the accuracy of clinical models are standard given in confidence intervals at least using typical statistical indicators: sensitivity, specificity, PPV and NPV. For better reception, it is worth listing them in the table.

Is the work clearly and accurately presented and does it cite the current literature?

Yes

If applicable, is the statistical analysis and its interpretation appropriate?

Partly

Are all the source data underlying the results available to ensure full reproducibility?

No

Is the study design appropriate and is the work technically sound?

No

Are the conclusions drawn adequately supported by the results?

Partly

Are sufficient details of methods and analysis provided to allow replication by others?

No

Reviewer Expertise:

computer vision, deep learning, reinforcement learning, biomedical engineering, parallel processing

I confirm that I have read this submission and believe that I have an appropriate level of expertise to confirm that it is of an acceptable scientific standard, however I have significant reservations, as outlined above.

# Response

**Published:** 2026-01-25

**Authors:** Alida Widiawaty

**Affiliations:** Universitas Riau, Pekanbaru, Riau, Indonesia

Dear Tomasz Krzywicki

 University of Warmia and Mazury in Olsztyn, Olsztyn, Warmian-Masurian Voivodeship, Poland 

 We thank the reviewer for the thorough evaluation of our manuscript and for the constructive comments that have substantially improved the clarity, transparency, and methodological rigor of the study. All points raised have been carefully addressed, and the manuscript has been revised accordingly.

**1. Clarification of clinical information**

- Reviewer comment: It is worth specifying at the beginning what kind of clinical information was included in the text data. Was there information that was unobservable from the point of view of the visual skin analysis, which meant that it was not correlated with the information contained in the photos?
- Response: Thank you for this valuable suggestion. We have clarified the clinical variables included in the textual modality at the beginning of the Methods section. Specifically, clinical information incorporated into the text data for non–atopic dermatitis (non-AD) cases comprised sex, age, chief complaint with onset, history of exposure to allergens or irritants, source of infection, current triggering factors, duration of illness, lesion location, past medical history, and family medical history. For atopic dermatitis (AD) cases, additional inputs included major and minor diagnostic criteria. Importantly, several of these variables—such as symptom onset, exposure history, triggering factors, and family history—are not directly observable from visual skin analysis and therefore provide complementary, non-visual clinical context beyond the information contained in the images.

**2. Clarification of the specialists' experience**

- Reviewer comment: It is worth clarifying what experience the specialists have had in describing the data.
- Response: Thank you for this suggestion. We have clarified the expertise of the annotators in the Methods section. Specifically, all images were independently annotated by two board-certified dermatologists, each with over 20 years of clinical experience in inflammatory dermatoses, achieving 100% inter-rater agreement.

**3. Clarification of how the photo descriptions was selected**

- Reviewer comment: Is it worth clarifying how the ground truth of the photo descriptions was selected? What would be the procedure if the specialists (with a completely independent evaluation) assigned different ratings to the photos?
- Response: Thank you for this comment. Ground truth photo descriptions were established based on independent annotations performed by two board-certified dermatologists. Complete inter-rater agreement (100%) was achieved across all images; therefore, all annotated photo descriptions were retained for analysis. We have added clarification that, in the event of discrepant ratings, images with discordant annotations and their associated clinical data would have been excluded from the dataset to avoid introducing ambiguity in the ground truth labels.

**4. Revision of the results section**

- Reviewer comment: Sections regarding descriptions of dataset characteristics, details of training models and initial data preparation should be moved from "Results" to "Methods" or create a separate chapter, e.g. "Materials". The details of the training do not apply to the results. This chapter should contain the final results.
- Response: Thank you for this comment. We have revised the manuscript structure by moving the descriptions of dataset characteristics, model training details, and initial data preparation from the Results section to a separate chapter entitled Materials, as suggested. The Results section has been streamlined to present only the final performance outcomes of the models.

**5. Clarification of the comparison between ResNet and Vision Transformer (ViT)**

- Reviewer comment: Table 2 should be included in the paragraph with the first reference to. In this table, the authors present the result of the comparison of the accuracy of the classification obtained on the ViT and ResNet architectures. It is worth mentioning that these results, with the current data set, should not be summarised with each other. One of the important requirements of the Transformer architecture is a sufficiently large set of training data, where most often the minimum requirements apply to several hundred thousand images, and reasonably, at least a million. With such a small dataset, the ViT architecture is doomed to failure. With such a small dataset, other CNN-based architectures should be compared, e.g. ResNet, Inception and Xception.

- Response: Thank you for this valuable comment. Table 2 has been relocated to appear immediately after its first mention in the text. In response to the reviewer’s concern, the direct performance comparison with the Vision Transformer (ViT) architecture has been removed from Table 2. We have also revised the *Methods* section to clarify that the ViT model was included for exploratory purposes only. Given the limited dataset size, ViT was not expected to achieve optimal performance, as transformer-based architectures generally require substantially larger training datasets. Accordingly, ViT results were not used as a basis for direct performance comparison with CNN-based models. In addition, the *Discussion* section has been expanded to explicitly state that any comparison between CNN-based architectures and ViT should be interpreted with caution. The relatively small dataset in this study likely constrained the performance of the ViT model, consistent with the known data requirements of transformer-based approaches.

**6. Clarification of the final multimodal architecture and information about the training**

- Reviewer comment: The article lacks a coherent scheme presenting the final multimodal architecture and information about the training. Yes, Figure 3 presents a complete flow from data collection stage to inference. However, there is no information about the final model itself. Were the models that process the image and text learned together with the final classifier, jointly correcting errors? Or is the final classifier a separate model that classifies independent image and text inputs?
- Response: Thank you for this comment. We have clarified the final multimodal architecture and training strategy in the Methods section. Once ResNet50 and MPNet processed their respective modalities and generated modality-specific feature embeddings, the resulting vectors were concatenated to form a unified multimodal representation. This late-fusion strategy integrates fine-grained visual information from images with contextual information derived from clinical text. The image and text encoders were trained independently, and their parameters were kept fixed during multimodal training. The concatenated feature vector was subsequently passed through several fully connected layers, which were trained as a separate classifier to distinguish between atopic dermatitis (AD) and non–atopic dermatitis (non-AD) cases. No end-to-end joint optimization across modalities was performed.

**7. Revision of results section and removal of redundant figures**

- Reviewer comment: Figures 1 and 2 only provide information about how the quality of the classification indicators changed during the training iterations. In my opinion, they do not contribute anything significant to the results. In addition, Figure 1 shows the features of overfitting model based on the ResNet architecture, which raises serious doubts about the reliability of the results.
- Response: Thank you for this insightful comment. We agree that Figures 1 and 2 primarily illustrate training dynamics rather than final model performance and do not add substantial value to the presentation of the results. Moreover, as the learning curves may raise concerns regarding overfitting, we have removed Figures 1 and 2 from the revised manuscript. The Results section has been refocused on the final evaluation metrics obtained on the test set, which more accurately reflect the clinical performance of the proposed models.

**8. Handling Class Imbalance and Evaluation Metrics**

- Reviewer comment: The dataset used in the experiment was unbalanced in terms of the number of photos in decision classes. The authors do not specify how they solved this problem in the context of training and evaluating model results. According to the names of the quality measures, the authors refer to their global versions intended for balanced datasets.
- Response:  Thank you for this comment. The dataset was imbalanced with respect to the number of samples per class. To address this issue during model development, we have added information indicating that stratified splitting was applied to preserve class proportions across the training, validation, and test sets. For performance evaluation, class-imbalance–aware metrics were used, including weighted precision, weighted recall, and weighted F1-score, in addition to overall accuracy. These weighted metrics compute class-wise scores and aggregate them according to class prevalence, thereby providing a more reliable assessment under imbalanced data conditions.

**9. Inclusion of research flow diagrams**

- Reviewer comment: Figures 4 and 5 are trivial and contribute nothing but to the repetition of the content of the previous paragraphs.
- Response: Thank you for this comment. Figures 2 and 3 (previously Figures 4 and 5) were included to comply with the STARD 2015 reporting guidelines, specifically the recommended flow of participants diagram. These figures provide a transparent visual summary of the study flow, including participant inclusion and data processing steps, which may not be fully conveyed through narrative text alone.

**10. **Addition of confidence intervals and diagnostic performance table****

- Reviewer comment: The final results of the accuracy of clinical models are standard given in confidence intervals at least using typical statistical indicators: sensitivity, specificity, PPV and NPV. For better reception, it is worth listing them in the table.

- Thank you for this important comment. We have revised the manuscript to report the final diagnostic performance of the multimodal ResNet50–MPNet model using standard clinical metrics, including accuracy, sensitivity, specificity, PPV, and NPV, each accompanied by 95% confidence intervals. The final accuracy of 98.28% (95% confidence interval = 96.93 - 99.65), sensitivity of 99.71% (95% confidence interval = 99.14 - 100), specificity of 97.21% (95% confidence interval = 95.01 - 99.41), Negative Predictive Value (NPV) 99.5% and Positive Predictive Value (PPV) 98.3%. These results are now summarized in a dedicated table entitled *“Diagnostic Performance of the Multimodal ResNet50–MPNet Model”* to improve clarity and clinical interpretability.

We have revised the manuscript in accordance with all the reviewers’ comments. Thank you once again for your constructive guidance. Please let us know if any further clarification or revisions are required.

 Warm regards,

 Alida Widiawaty

 Faculty of Medicine, Universitas Riau, Pekanbaru, 28133, Indonesia

 The Doctoral Program in Medical Sciences, Faculty of Medicine Universitas Indonesia, Jakarta, 10430, Indonesia

# Reviewer report: Reviewer response for version 1

**DOI:** 10.5256/f1000research.186388.r420623

**Published:** 2025-10-29

**Authors:** Sebastian Sitaru

**Affiliations:** 1 Technical University of Munich, Munich, Munich, Germany

In their manuscript, the authors describe the creation and validation of a multimodal image-text AI algorithm for the diagnosis of atopic dermatitis.

 The topic is highly relevant and the technical approach seems sound. The manuscript is overall well written and concise. It is commendable that the authors decided to share their source code.

 I have a couple of minor points which could be improved before Indexing:

 - In the methods it says, "Two board-certified dermatologists independently annotated all images". Do you mean all cases, so image+text? Or did they just annotate the images alone?

 - Was the skin tone of the patients recorded? Even if not, I think this point deserves a mention in the discussion

Is the work clearly and accurately presented and does it cite the current literature?

Yes

If applicable, is the statistical analysis and its interpretation appropriate?

Yes

Are all the source data underlying the results available to ensure full reproducibility?

Yes

Is the study design appropriate and is the work technically sound?

Yes

Are the conclusions drawn adequately supported by the results?

Yes

Are sufficient details of methods and analysis provided to allow replication by others?

Yes

Reviewer Expertise:

Data analysis and AI applications in dermatology

I confirm that I have read this submission and believe that I have an appropriate level of expertise to confirm that it is of an acceptable scientific standard.

# Response

**Published:** 2026-01-25

**Authors:** Alida Widiawaty

**Affiliations:** Universitas Riau, Pekanbaru, Riau, Indonesia

Dear Sebastian Sitaru

 Technical University of Munich, Munich, Germany

 We thank the reviewer for the thorough evaluation of our manuscript and for the constructive comments that have substantially improved the clarity, transparency, and methodological rigor of the study. All points raised have been carefully addressed, and the manuscript has been revised accordingly.

**1. Clarification of annotation scope **

- Reviewer comment: In the methods it says, "Two board-certified dermatologists independently annotated all images". Do you mean all cases, so image + text? Or did they just annotate the images alone?
- Response: Thank you for this insightful comment. We have clarified this point in the manuscript to state that two board-certified dermatologists independently annotated both the clinical images and the corresponding text data for all cases.

**2. Clarification of patients' skin tone**

- Reviewer comment: Was the skin tone of the patients recorded? Even if not, I think this point deserves a mention in the discussion
- Response:  Thank you for this comment. Skin tone was not explicitly recorded as a standalone variable in this study. However, the study population predominantly consisted of Indonesian patients, who generally fall within Fitzpatrick skin phototypes III–IV. Within this relatively homogeneous skin tone range, no systematic variation in clinical presentation or image-based model performance attributable to skin tone was observed. Therefore, skin tone was not considered a confounding factor influencing the study outcomes. Nonetheless, we acknowledge in the Discussion that external validation in cohorts with a broader range of Fitzpatrick skin types would be valuable to confirm the model’s robustness across more diverse populations.

We have revised the manuscript in accordance with all the reviewers’ comments. Thank you once again for your constructive guidance. Please let us know if any further clarification or revisions are required.

 Warm regards,

 Alida Widiawaty

 Faculty of Medicine, Universitas Riau, Pekanbaru, 28133, Indonesia

 The Doctoral Program in Medical Sciences, Faculty of Medicine Universitas Indonesia, Jakarta, 10430, Indonesia
